# Effects of Optical Sampling Pulse Power, RF Power, and Electronic Back-End Bandwidth on the Performance of Photonic Analog-to-Digital Converter

**DOI:** 10.3390/mi14122155

**Published:** 2023-11-25

**Authors:** Junli Qi, Xin Chen, Meicheng Fu, Hongyu Zhang, Wenjun Yi, Tengfei Xu, Dezhi Su, Hui Zhang, Xiaoming Wei, Bo Shi, Xiujian Li

**Affiliations:** 1 College of Science, National University of Defense Technology, Changsha 410073, China; qijunli_r@nudt.edu.cn (J.Q.); chenxin_cx@nudt.edu.cn (X.C.); fumeicheng10@nudt.edu.cn (M.F.); zhanghongyu@nudt.edu.cn (H.Z.); yiwenjun@nudt.edu.cn (W.Y.); 2 Basic Department, Army Academy of Artillery and Air Defense, Hefei 230031, China; yhhzhhf@163.com (H.Z.); shibo1982_1982@126.com (B.S.); 3 Beijing Institute of Systems Engineering and Information Control, Beijing 100071, China; xtf1201@126.com; 4 College of Basic Sciences for Aviation, Naval Aviation University, Yantai 264001, China; sudezhifun@163.com

**Keywords:** photonic analog-to-digital converter, optical sampling pulse power, RF power, electronic back-end bandwidth, signal recovery, microwave photonics

## Abstract

The effects of optical sampling pulse power, RF power, and electronic back-end bandwidth on the performance of time- and wavelength-interleaved photonic analog-to-digital converter (PADC) with eight-channel 41.6 GHz pulses have been experimentally investigated in detail. The effective number of bits (ENOB) and peak-to-peak voltage (*V_pp_*) of converted 10.6 GHz electrical signals were used to characterize the effects. For the 1550.116 nm channel with 5.2 G samples per second, an average pulse power of 0 to −10 dBm input to the photoelectric detector (PD) has been tested. The *V_pp_* increased with increasing pulse power. And the ENOB for pulse power −9~−3 dBm was almost the same and all were greater than four. Meanwhile, the ENOB decreased either when the pulse power was more than −2 dBm due to the saturation of PD or when the pulse power was less than −10 dBm due to the non-ignorable noise relative to the converted weak signal. In addition, RF powers of −10~15 dBm were loaded into the Mach–Zehnder modulator (MZM). The *V_pp_* increased with the increase in RF power, and the ENOB also showed an increasing trend. However, higher RF power can saturate the PD and induce greater nonlinearity in MZM, leading to a decrease in ENOB, while lower RF power will convert weak electrical signals with more noise, also resulting in lower ENOB. In addition, the back-end bandwidths of 0.2~8 GHz were studied in the experiments. The *V_pp_* decreased as the back-end bandwidth decreased from 8 to 3 GHz, and remained nearly constant for the bandwidth between the Nyquist bandwidth and the subsampled RF signal frequency. The ENOB was almost the same and all greater than four for a bandwidth from 3 to 8 GHz, and gradually increased up to 6.5 as the back-end bandwidth decreased from the Nyquist bandwidth to 0.25 GHz. A bandwidth slightly larger than the Nyquist bandwidth was recommended for low costs and without compromising performance. In our experiment, the −3 to −5 dBm average pulse power, about 10 dBm RF power, and 3 GHz back-end bandwidth were recommended to accomplish both a high ENOB more than four and large *V_pp_*. Our research provides a solution for selecting optical sampling pulse power, RF power, and electronic back-end bandwidth to achieve low-cost and high-performance PADC.

## 1. Introduction

Compared to analog signals, digital signals have many advantages such as good stability, strong anti-interference ability, easy recording, storage, and editing, and good confidentiality, while the analog-to-digital converter (ADC) is a key device for converting analog signals into digital signals. In the process of information processing, ADC plays a very important role, especially in the cutting-edge technology fields of large bandwidth and ultra-high-speed microwave signal processing systems [1,2,3,4,5]. Due to limitations such as large time jitter, large high-frequency loss, and comparator ambiguity, the traditional electric ADC (EADC) struggles to meet the requirements of high sampling rates and large bandwidths.

Photonic ADC (PADC) based on ultra-short optical pulse trains has been demonstrated to be a powerful tool to achieve an ultra-high sampling rate, ultra-wideband bandwidth, and to overcome the bottleneck of electric ADC [6,7]. Among various kinds of PADC schemes, the photonic-sampled and electronic-quantized PADC, which takes advantage of photons in wideband processing and electrons in high-precision quantization, has become one of the mainstream schemes [8,9,10,11,12,13,14]. For instance, using a time- and wavelength-interleaved optical sampling pulse train, Qingwei Wu and their team realized a PADC with a 40 GS/s sampling rate. A 2.5 GHz sinusoidal RF signal was sampled and quantized using the PADC system, achieving the effective number of bits (ENOB) of 3.45 bits [8]. A two-channel PADC system with a 10 GHz repetition rate and 19.8 fs time jitter pulse source was implemented by Zhengkai Li and their team, successfully sampling and quantifying RF signals of 1.1 GHz and 36.3 GHz into ENOBs of 5.85 and 3.75 bits, respectively [15]. In this PADC scheme, RF signals are sampled through optical pulses and digitized through electronic quantizers, such as EADC and oscilloscope. Thanks to the high bandwidth and fast response of the electro-optic modulator, high-frequency RF signals can be directly loaded into high-speed optical sampling pulses smoothly without multichannels, while the sampled pulses carrying RF signals need to be demultiplexed to multiple channels to adapt to EADC with a low sampling rate. This kind of PADC is collectively referred to as channel-interleaved PADC (CI-PADC). Usually, based on the method of demultiplexing, CI-PADC can be divided into time-division demultiplexed PADC [16,17,18,19] and time- and wavelength-interleaved PADC [20,21]. Of course, both methods can also be used simultaneously [7].

Many studies have been conducted on the influence of channel mismatch, including amplitude mismatch, delay mismatch, pulse shape mismatch, and electronic aperture jitter, on the performance of PADC [16,17,18,22,23], while there are few research reports on the effects of optical sampling pulse power, RF power, and electronic back-end bandwidth. In this paper, the effects of 0~−10 dBm optical pulse power, −10~15 dBm RF power, and 0.2~8 GHz electronic back-end bandwidth on the performance of time- and wavelength-interleaved PADC with eight-channel 41.6 GHz pulses have been experimentally investigated in detail. The 10.6 GHz RF signals were loaded into the PADC, and the ENOB and peak-to-peak voltage (*V_pp_*) were calculated and discussed. Our research provides a solution for choosing pulse power, RF power, and electronic back-end bandwidth to achieve low-cost and high-performance PADC.

## 2. Data Processing and Signal Recovery Method

For high-speed PADC, the sampling rate (SR), signal-to-noise and distortion (SINAD) ratio, effective number of bits (ENOB), and spurious-free dynamic range (SFDR) of the system have attracted more attention. Among them, SR and SFDR are relatively intuitive and easy to obtain directly from experimental data, while the three-parameter least square fit method is usually used to obtain SINAD and ENOB [24].

Firstly, the output sequence of the ADC is recorded, which includes M sampling points *y*_1_, *y*_2_, …, and *y_M_* recorded at *t*_1_, *t*_2_, …, and *t_M_*. Use the algorithm of the following matrix to find *A*, *B*, and *C* so that the sum of variances in Equation (1) is minimized.

(1) $$\sum_{n=1}^{M}{\left[y_{n}-A\cos(\omega_{0}t_{n})-B\sin(\omega_{0}t_{n})-C\right]}^{2},$$

in which *ω*_0_ is the input signal frequency loaded in the ADC.

In order to find the values of *A*, *B*, and *C*, matrices *D*_0_, *y*, and *x* are created as follows:

(2) $$D_{0}=\left[\begin{array}{lll} \cos(\omega_{0}t_{1}) & \sin(\omega_{0}t_{1}) & 1\\ \cos(\omega_{0}t_{2}) & \sin(\omega_{0}t_{2}) & 1\\ \vdots & \vdots & \vdots \\ \cos(\omega_{0}t_{M}) & \sin(\omega_{0}t_{M}) & 1 \end{array}\right],\;y=\left[\begin{array}{c} y_{1}\\ y_{2}\\ \vdots \\ y_{M} \end{array}\right],\;x=\left[\begin{array}{c} A\\ B\\ C \end{array}\right]$$

From a matrix perspective, the sum of variances in Equation (1) can be transformed into the following equation

(3) $${\left(y-D_{0}x\right)}^{T}(y-D_{0}x)$$

Minimizing the Equation (3), the minimum square solution is calculated by

(4) $$x={\left(D_{0}{}^{T}D_{0}\right)}^{-1}\cdot (D_{0}{}^{T}y)$$

The fitted function can be expressed as follows:

(5) $$y_{n}^{\prime }=A\cos(\omega_{0}t_{n})+B\sin(\omega_{0}t_{n})+C,$$

which is also the recovered signal function.

The residual *r_n_* after fitting can be expressed as follows:

(6) $$r_{n}=y_{n}-y_{n}^{\prime }=y_{n}-A\cos(\omega_{0}t_{n})+B\sin(\omega_{0}t_{n})+C$$

Then, the RMS value of noise can be calculated by

(7) $$V_{rms\_noise}={\left[\frac{1}{M}\sum_{n=1}^{M}{\left(r_{n}\right)}^{2}\right]}^{1/2}$$

And the RMS value of the signal can be expressed as follows:

(8) $$V_{rms\_signal}=(V_{pp}/2)/\sqrt{2},$$

in which *V_pp_* is the peak-to-peak value of the recovered sinusoidal signal.

Thus, the *SINAD* can be obtained by the following equation:

(9) $$SINAN=20\times \log_{10}(\frac{V_{rms\_noise}}{V_{rms\_signal}})$$

And the *ENOB* can be calculated by the following equation:

(10) ENOB=(SINAN−1.76)/6.02

The above are the recovery results of RF signals loaded in the ADC system, as well as the data processing methods for *SINAD* and *ENOB*.

## 3. Experimental Setup

**Figure 1:**
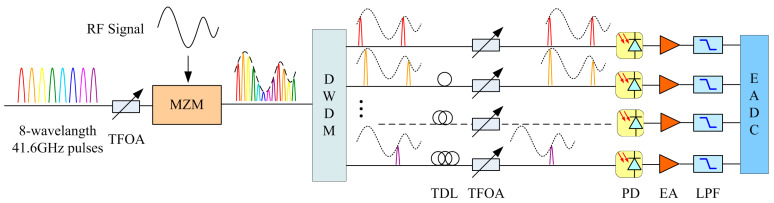
Eight-channel 41.6 GHz time- and wavelength-interleaved PADC system sketch. TFOA, tunable fiber-optic attenuator; MZM, Mach–Zehnder modulator; DWDM, dense wavelength division multiplexer; TDL, tunable delay line; PD, photoelectric detector; EA, electric amplifier; LPF, low-pass filter.

The time- and wavelength-interleaved PADC system is depicted in Figure 1. Equally spaced, aligned eight-wavelength 41.6 GHz sampling pulse sources can be generated by suitable modulation of cascaded intensity modulators and phase modulators to continuous-wave lasers [25,26,27]. The tunable fiber-optic attenuator (TFOA) is used to adjust optical sampling pulse power loaded into the Mach–Zehnder modulator (MZM) with broad bandwidth, making sure it is working in a good condition. Meanwhile, the RF signals are loaded onto sampling pulse sources by the MZM. The optical pulses loaded with RF signals are demultiplexed by dense wavelength division multiplexer (DWDM) and then converted into paralleled eight-channel single-wavelength 5.2 GHz optical signals. Each channel is processed separately. Due to the possible delay changes in different wavelengths after DWDM, the eight-channel optical pulses are synchronized again through a tunable delay line (TDL). In addition, due to the different insertion losses of different wavelengths and fiber lengths caused by DWDM, the optical intensity is relatively compensated through the TFOA, which also makes sure the optical pulse intensity is located in the linear working area of the photoelectric detector (PD). Then, the optical signal is converted into an electrical signal through PD and is amplified by the electric amplifier (EA) and limited with proper bandwidth through a low-pass filter (LPF) to put it in the optimal working state of the EADC with the benefits of mature production process, stable performance, and high quantization accuracy at appropriate transmission rates.

In our experiment, the eight central wavelengths from long to short are 1557.363, 1554.940, 1552.524, 1550.116, 1547.715, 1545.322, 1542.936, and 1540.577 nm, respectively. The MZM has a 3-dB bandwidth of 28G, ensuring the ability to load broad bandwidth RF signals. The PD has a bandwidth of 10 GHz with built-in electric amplification. The EADC is substituted by Tektronix’s MSO64 oscilloscope, which has a bandwidth of 8 GHz, an equivalent sampling rate of up to 2.5 T samples per second (TSa/s), and a sampling time interval of 0.4 ps. For each 5.2 GHz pulse, the oscilloscope will collect approximately (2.5T/5.2G) ≈ 481 sampling points, among which the maximum value will be selected as the sampling quantization value of the pulse.

In order to investigate the effects of pulse power, radio frequency power, and quantization bandwidth on the performance of the entire PADC system, each channel must be researched separately. However, for each channel, all device compositions are the same, which means that all channels have similar patterns. Thus, we only need to study the influence law of one channel, and the channel corresponding to 1550.116 nm was selected as our research object. In order to verify the broad bandwidth of PADC, a 10.6 GHz RF signal higher than the 8 GHz bandwidth of the oscilloscope was selected in the experiment.

## 4. Results and Discussion

Experimental research and analyses were conducted on the effects of different input optical sampling pulse powers, RF powers ranging from −10 to 15 dBm, and different electronic back-end bandwidths on the performance of PADC. The ENOB and *V_pp_* of converted RF signal were used to characterize the effects.

### 4.1. Effect of Optical Sampling Pulse Power

**Figure 2:**
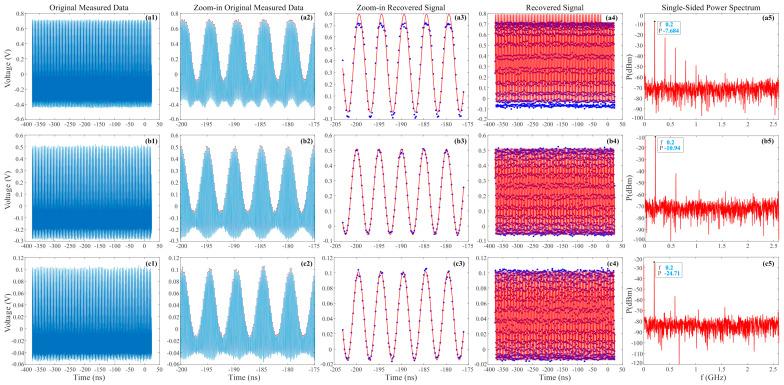
Results of the oscilloscope collecting and processing 10.6 GHz RF signal with 10 dBm power by the PADC with different average pulse power input. (**a**–**c**) Situations with average pulse power of 0, −3, −10 dBm input to the PD, respectively; (**1**) original measured 1M data; (**2**) zoom-in original measured data; (**3**) zoom-in recovered signal; (**4**) all recovered signal; (**5**) single-sided power spectra.

The MZM in the PADC is modulated to operate at the quadrature work point. The results of the oscilloscope collecting and processing 10.6 GHz RF signal with 10 dBm power by the PADC with different average pulse power input to MZM are shown in Figure 2. According to the Nyquist sampling theorem, the sampling bandwidth corresponding to a 5.2 GHz pulse is 2.6 GHz, and for the 10.6 GHz signal, it will be subsampled to 200 MHz. Figure 2a–c correspond to the situations when the average pulse power of pulses modulated by MZM input to the PD is 0, −3, and −10 dBm, respectively, in which the maximum allowable optical power for the PD input shall not exceed 10 dBm. Figure 2(a1–c1) show all original data of 1M sampling points collected by the oscilloscope. It is found that the larger the input pulse power, the higher the maximum value of the collected signal and the lower the minimum value. However, due to the large amount of data, detailed information is missing. Overall, they seem to have the same pattern and it is hard to see the difference. The zoom-in original data for approximately five cycles with more details are depicted in Figure 2(a2–c2), where the maximum value of each pulse is marked with a red dot. It can be observed that Figure 2(a2) shows a flat top, indicating that excessive energy input can make the PD saturated. And the trigonometric envelope has been clearly shown in Figure 2(b2,c2). Figure 2(a3–c3) show the zoom-in recovered signal with a red line in the method of Part 2, and the maximum measured value of each pulse is displayed with a blue dot. The recovered red signal is higher than the measured data in Figure 2(a3) and a little lower in Figure 2(c3), and the recovered signal fits well with the measured values in Figure 2(b3). The recovered signals of all maximum measured values are shown in Figure 2(a4–c4), which more clearly depict the same law as Figure 2(a3–c3). It is particularly evident that the measured blue data is higher than the recovered red signal in Figure 2(c4).

Figure 2(a5–c5) depict the single-sided power spectra between 0 and 2.6 GHz by the FFT of all collected maximum values in every pulse. And the fundamental frequency and corresponding power value are marked in the figure. It can be observed that the higher the input optical power, the greater the fundamental frequency power obtained. More high-energy spectra, except for the fundamental frequency, can be found in Figure 2(a5), further indicating that high-energy optical pulse input will lead to significant nonlinearity. Comparing Figure 2(b5,c5), it can be found that the fundamental frequency energy of Figure 2(b5) is 13.77 dB higher than that of Figure 2(c5), while the noise level of Figure 2(c5) (approximately −82 dBm) is only about 10 dB lower than that of Figure 2(b5) (approximately −72 dBm), indicating that the signal corresponding to Figure 2(c5) has lower signal-to-noise ratio, i.e., lower power pulses input will reduce the signal-to-noise ratio.

**Figure 3:**
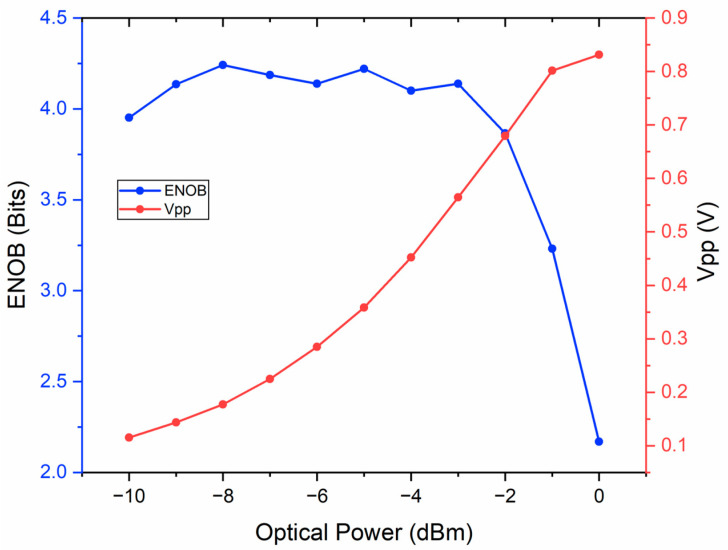
Measured ENOB and *V_pp_* with the input average pulse power from 0 to −10 dBm to PD.

Figure 3 shows the calculated ENOB using measured 1M data and the peak-to-peak voltage *V_pp_* of recovered signals with the average pulse power input to PD gradually decreasing from 0 to −10 dBm modulated by TFOA. It can be found that the ENOB for pulse power −9~−3 dBm is almost the same and all greater than four. And ENOB will decrease either when the pulse power is more than −2 dBm due to the saturation of PD, or when the pulse power is less than −10 dBm due to the non-ignorable noise relative to the converted weak signal. The *V_pp_* increases with increasing pulse power. In order to meet both high ENOB and large *V_pp_* requirements, the average pulse power of −3~−5 dBm input to PD is suggested.

### 4.2. Effect of RF Power

**Figure 4:**
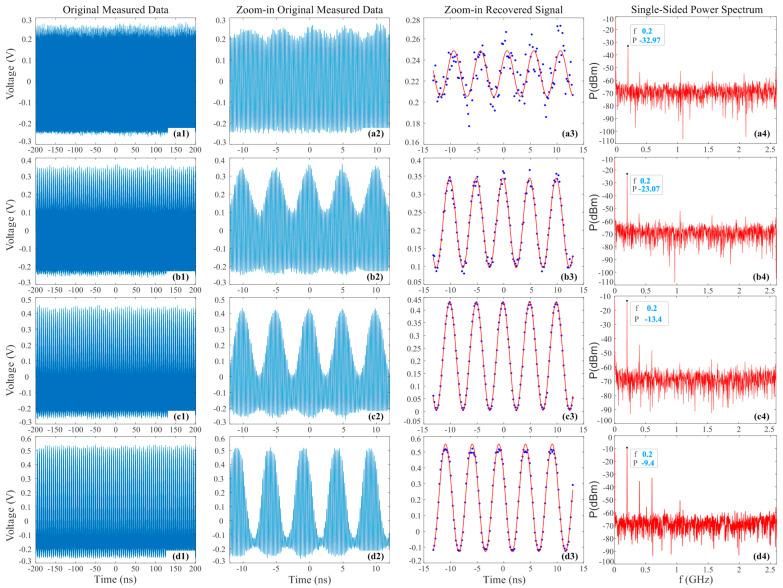
Results of the oscilloscope collecting and processing 10.6 GHz RF signal with −10 (**a**), 0 (**b**), 10 (**c**), and 15 (**d**) dBm power by the PADC with −4 dBm average pulse power input to PD; (**1**) original measured 1 M data; (**2**) zoom-in original measured data; (**3**) zoom-in recovered signal; (**4**) single-sided power spectra.

Figure 4 shows the results of the oscilloscope collecting and processing 10.6 GHz RF signal with different power by the PADC with −4 dBm average pulse power input to PD. Figure 4a–d are the situations for RF signal of −10, 0, 10, and 15 dBm power, respectively. It was found that the minimum values measured by the oscilloscope were almost the same due to the same optical sampling pulse power. The larger the RF power, the higher the measured maximum value, and the corresponding peak-to-peak value *V_pp_* of the recovered signal is also larger. For low RF power, the measurement noise has a significant impact, while for high RF power, the measurement values exhibit a flat top due to large nonlinearity of MZM and saturation of PD. Among them, the recovered signal fit best with the measured values for the 10 dBm RF signal. Figure 4(a4–d4) depict the corresponding single-sided power spectrum. And the fundamental frequency and corresponding power value are marked in the figure. It could be found that the higher the loaded RF power, the greater the fundamental frequency power. In Figure 4(d4), it can be observed that there are more high-energy, high-frequency components in addition to the fundamental frequency, indicating that loading an RF signal with excessive energy will lead to significant nonlinearity.

**Figure 5:**
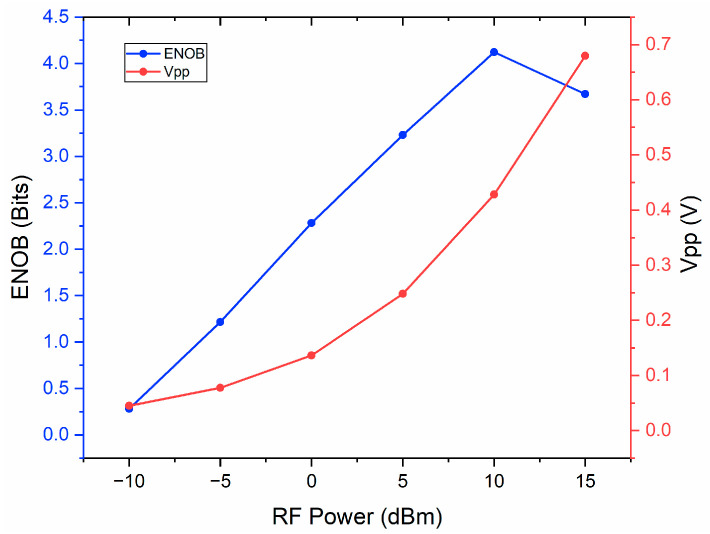
Measured ENOB and *V_pp_* of recovered signal with the input RF power from −10 to 15 dBm.

The measured ENOB and *V_pp_* of the recovered signal with the input RF power of −10, −5, 0, 5, 10, and 15 dBm were shown in Figure 5. It was found that with the increase in RF power, the *V_pp_* increases correspondingly, and the ENOB also shows an increasing trend. However, higher RF power can saturate the PD and induce greater nonlinearity in MZM, leading to a decrease in ENOB. Overall, the RF signal with a power of 10 dBm has the best performance with a high ENOB greater than four and appropriate peak-to-peak value.

### 4.3. Effect of Electronic Back-End Bandwidth

**Figure 6:**
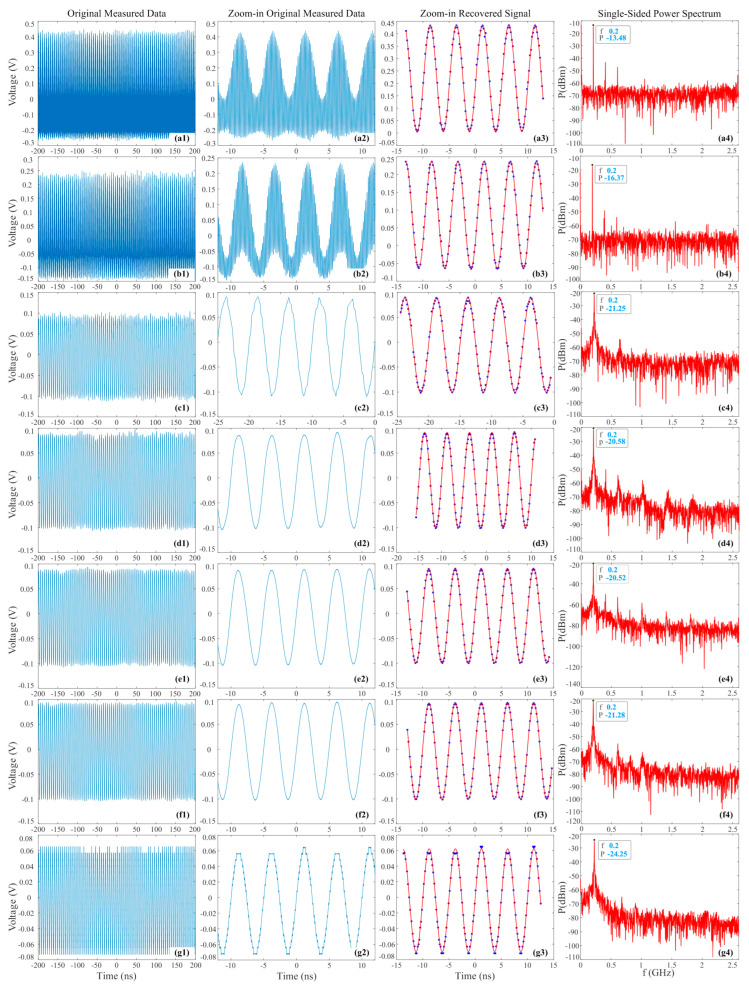
Results of the oscilloscope collecting and processing 10.6 GHz RF signal through the PADC filtered by LPFs with 8 (**a**), 5 (**b**), 3 (**c**), 1 (**d**), 0.5 (**e**), 0.25 (**f**), and 0.2 (**g**) GHz bandwidth, respectively; (**1**) original measured data; (**2**) zoom-in original measured data; (**3**) zoom-in recovered signal; (**4**) single-sided power spectra.

The electronic back-end bandwidth is a comprehensive one that includes the bandwidth of PD and EADC (or oscilloscope). Figure 6 shows the results of the oscilloscope collecting and processing the 10.6 GHz RF signal through the PADC filtered by LPFs with different bandwidths. The average pulse power input to PD is −4 dBm, and the RF power is 10 dBm. Figure 6a–g are the situations for LPFs of 8, 5, 3, 1, 0.5, 0.25, and 0.2 GHz bandwidth, respectively. It was found that for the back-end bandwidth of 8 GHz in Figure 6(a1–a4), an entire pulse shape of 5.2 GHz can be easily collected with the 2.5 TSa/s sampling rate. And for the 5 GHz bandwidth in Figure 6(b1–b4), some information for each pulse has been filtered out, resulting in a decrease in the maximum value and increase in the minimum value, but the contour of 5.2 GHz pulse can still be seen. However, for the bandwidth of 3 GHz and below, 5.2 GHz pulses were almost invisible, and only the maximum values of pulses were preserved. For the subsampled 10.6 GHz RF signal with 5.2 GHz pulses, when the bandwidth approaches 200 MHz from high to low, the signal contour measured by the oscilloscope became smoother, while when the bandwidth was 0.2 GHz and below, the collected signal contour was no longer smooth and appeared serrated. Therefore, the back-end bandwidth must be higher than the frequency of the subsampled RF signal. In addition, the recovered signal fitted very well with the measured values for the bandwidth greater than 0.25 GHz. The corresponding single-sided power spectrum were depicted in Figure 6(a4–d4). And the fundamental frequency and corresponding power value are marked in the figure. It can be observed that as the bandwidth decreased from 3 GHz to 8 GHz, the power of the fundamental frequency also decreased, while for the bandwidth between 3 GHz and 0.25 GHz, the fundamental frequency power remained basically unchanged. When the bandwidth was below 200 MHz, the fundamental frequency power would decrease rapidly when the fundamental component was filtered.

**Figure 7:**
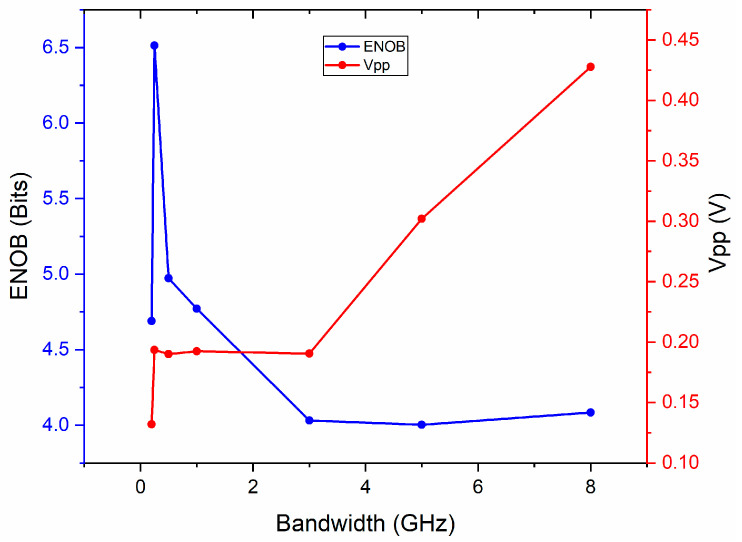
Measured ENOB and *V_pp_* o with electronic back-end bandwidth from 0.2 to 8 GHz.

Figure 7 shows the measured ENOB and *V_pp_* of the recovered signal with electronic back-end bandwidth from 0.2 to 8 GHz. It can be found that the ENOB for bandwidth from 3 to 8 GHz is almost the same and all greater than four. This was because, for a sampling rate of 5.2 GSa/s, the Nyquist bandwidth is 2.6 GHz. When the back-end bandwidth exceeds 2.6 GHz, complete sampling can be achieved and sampling performance is not reduced. In addition, as the back-end bandwidth decreased from the Nyquist bandwidth to 0.25 GHz, the ENOB gradually increased up to 6.5 bits. That was because the higher-order terms of the subsampled RF signal within the Nyquist bandwidth were filtered out. And it can be easily found that the ENOB and *V_pp_* will significantly decrease when the back-end bandwidth is lower than the subsampled RF signal frequency. In addition, the *V_pp_* decreased as the back-end bandwidth decreased from 8 to 3 GHz, and remained almost unchanged when the bandwidth was between the Nyquist bandwidth and the subsampled RF signal frequency. That is because only the peak energy of the pulse is collected within the Nyquist bandwidth. When the back-end bandwidth exceeds the Nyquist one, more parts of a single pulse are collected as the bandwidth increases, and the corresponding converted electrical energy also increases. For PD or EADC, the larger the bandwidth, the higher the price. Therefore, considering all possible subsampled frequencies, a bandwidth slightly larger than the Nyquist bandwidth, such as 3 GHz for 5.2 GSa/s sampling rate, is recommended for low costs and without compromising performance.

**Figure 8:**
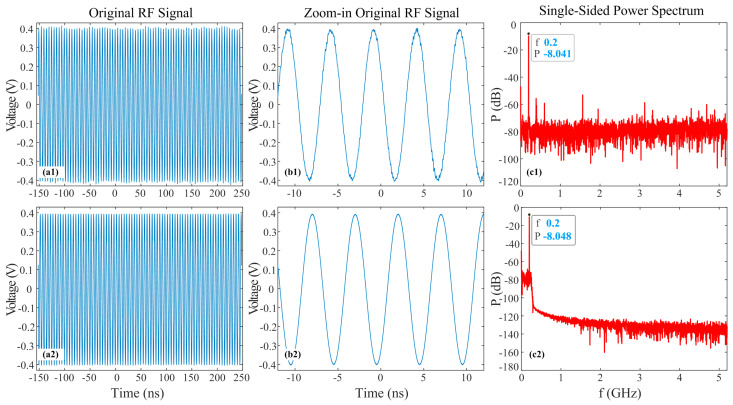
Original data and power spectra of 200 MHz RF signal in different bandwidth. (**a**) Original 1M RF signal data; (**b**) zoom-in original RF signal data; (**c**) single-sided power spectrum of RF signal; (**1**,**2**): 8 GHz and 0.25 GHz bandwidth.

In order to demonstrate that the oscilloscope has no impact on the quantization accuracy of the PADC system, the ENOB of the oscilloscope was measured. Taking the 200 MHz RF signal directly collected by the oscilloscope as an example, its ENOB was measured at different bandwidths from 0.2 to 8 GHz. The experimental results are shown in Figure 8 with the bandwidth of 8 GHz and 0.25 GHz, respectively. Figure 8(a1,a2) shows the 1M collected original RF signal, and the zoom-in original RF signal for approximately five cycles with more details is depicted in Figure 8(a2,b2). Figure 8(c1,c2) shows the single-sided power spectra of RF signals in the 5.2 GHz range instead of 1250 GHz to demonstrate more details. The calculated ENOB was up to 4.999 bits at 8 GHz bandwidth and 7.439 bits at 0.25 GHz bandwidth, surpassing the maximum ENOB of the PADC system at the corresponding bandwidth. Therefore, the oscilloscope did not limit the ENOB of the PADC.

## 5. Conclusions

In summary, we have conducted a detailed experimental study on the effects of optical sampling pulse power, RF power, and electronic back-end bandwidth on the performance of time- and wavelength-interleaved PADC with eight-channel 41.6 G samples per second (Sa/s). The EADC is substituted by Tektronix’s MSO64 oscilloscope with a sampling rate of up to 2.5 TSa/s. For the channel of 1550.116nm with 5.2 GHz pulses, the average pulse powers of 0 to −10 dBm input to the photoelectric detector (PD) have been tested; excessive pulse power will saturate PD and result in a flat top of the tested 10.6 GHz sinusoidal signal, which will induce a decrease in ENOB, and lower pulse power will lead to little peak-to-peak voltage (*V_pp_*) of the converted electrical signal, which will bring the decrease in signal-to-noise ratio. From the power spectra information, similar conclusions can be drawn. The average pulse power of −3~−5 dBm input to the given PD is suggested as it meets both a high ENOB more than four and large *V_pp_*. In addition, −10~15 dBm RF powers were loaded into the MZM. With the increase in RF power, the *V_pp_* correspondingly increases, and the ENOB also shows an increasing trend. Higher RF power will saturate PD and induce greater nonlinearity in MZM, leading to a decrease in ENOB, and lower RF power will convert a weak electrical signal with more noise. Overall, the 10 dBm power RF signal has the best performance with a high ENOB greater than four and appropriate *V_pp_*. In addition, the back-end bandwidths of 0.2~8 GHz were investigated in the experiment. The ENOB for bandwidth larger than the Nyquist one were almost the same. As the back-end bandwidth decreased from the Nyquist bandwidth to 0.25 GHz, the ENOB gradually increased up to 6.5 and the *V_pp_* remained almost unchanged. Considering all possible subsampling frequencies, a bandwidth slightly larger than the Nyquist bandwidth was recommended for low costs and without compromising performance. It should be noted that the recommended optical pulse power and RF power are applicable to the PD and MZM in our experiment, and there will be other optimized average pulse powers and RF powers for other devices. Our research provides a solution on how to select pulse power, RF power, and back-end bandwidth to achieve low-cost and high-performance PADC.

## Data Availability

Data are contained within the article.
